# Illumination matters Part III: Impact of light obstruction on illuminance from flexible ureteroscopes — a comparative PEARLS analysis

**DOI:** 10.1007/s00345-024-04910-9

**Published:** 2024-03-23

**Authors:** Jia-Lun Kwok, Eugenio Ventimiglia, Vincent De Coninck, Alba Sierra, Frédéric Panthier, Mariela Corrales, Yazeed Barghouthy, Vineet Gauhar, Benedikt Kranzbühler, Florian Alexander Schmid, Cédric Poyet, Daniel Eberli, Olivier Traxer, Etienne Xavier Keller

**Affiliations:** 1 Department of Urology, University Hospital Zurich, University of Zurich, Frauenklinikstrasse 10, 8091 Zurich, Switzerland; 2 Department of Urology, Tan Tock Seng Hospital, Singapore, Singapore; 3 Progressive Endourological Association for Research and Leading Solutions (PEARLS), Paris, France; 4 Endourology and Urolithiasis Working Group, Young Academic Urologists (YAU), Arnhem, The Netherlands; 5 Division of Experimental Oncology/Unit of Urology, Urological Research Institute, IRCCS Ospedale San Raffaele, Milan, Italy; 6 Department of Urology, AZ Klina, Brasschaat, Belgium; 7 Urology Department, Hospital Clinic de Barcelona, Villarroel 170, 08036 Barcelona, Spain; 8 GRC no 20, Groupe de Recherche Clinique sur la Lithiase Urinaire, Sorbonne Université, Hôpital Tenon, 75020 Paris, France; 9 Department of Urology, Centre Hospitalier de Valenciennes, Valenciennes, France; 10 Department of Urology, Ng Teng Fong General Hospital, Singapore, Singapore

**Keywords:** Flexible ureteroscopy, Obstruction, Background illuminance, Saline, Skew

## Abstract

**Purpose:**

Artifacts from poor ureteroscopes’ light design with shadowing and dark areas in the field of view have been reported. The aim was to quantify effects of light obstruction in a kidney calyx model.

**Methods:**

We evaluated a series of contemporary flexible ureteroscopes including the Storz Flex-Xc and Flex-X2s, Olympus V3 and P7, Pusen 7.5F and 9.2F, as well as OTU Wiscope using an enclosed 3D-printed pink in vitro kidney calyx model submerged in saline, where the field of light was intentionally partially obstructed alternatively at 12, 3, 6, and 9 o’clock. A color spectrometer was used for illuminance measurements at a 45° opening position in the background of the model.

**Results:**

Overall and mean background illuminance for each obstructive situation were significantly different between scopes for both 50% and 100% brightness settings (ANOVA *p* < 0.001). At 50% brightness setting, almost all scopes had their highest and lowest background illuminance with the 6 o’clock and 3 o’clock obstructive situation, respectively. At 100% brightness setting, these became 6 o’clock and 12 o’clock obstructive situations. Considering each obstructive situation individually, the Flex-Xc was consistently the scope with highest background illuminance and the Pusen 7.5F the lowest. Background illuminance for each obstructive situation varied significantly for each scope individually, with the greatest range of variability for Pusen 7.5F and V3.

**Conclusions:**

Illuminance performance of ureteroscopes within an obstructed calyx model differ significantly for various obstructive situations. Urologists should be aware of this to help guide their choice of ureteroscope.

**Supplementary Information:**

The online version contains supplementary material available at 10.1007/s00345-024-04910-9.

## Introduction

Ureteroscopy has been established as the most frequent intervention for renal stones in industrialized countries [1]. It has a gamut of diagnostic and therapeutic applications [2, 3]. Image quality in ureteroscopy depends on many factors [4], including the illuminance of light that falls onto the target field.

Undesirable shadowing in the endoscopic field of view from poor light source design has been reported [5]. Another source of obstruction to the light source is from the collecting system itself. This may particularly occur in situations where soft tissue or commonly the calyceal neck partially blocks the light in calyces that are difficult to reach. To the best of our knowledge, prior studies have not explored such obstructing light situations [6–12]. In addition, it has not yet been evaluated to what extent the recently described variability in illuminance intensity and skew may impact on such obstructive situations [13, 14].

The aim of the present study was to investigate the effects of partial obstruction of the light source(s) on background illuminance of several different ureteroscopes submerged in saline.

## Materials and methods

We evaluated a series of currently available flexible ureteroscopes at our institution: the Flex-Xc and Flex-X2s (Karl Storz SE & Co. KG, Tuttlingen, Germany), URF-P7 and URF-V3 (Olympus, Center Valley, PA, USA), Uscope 7.5F PU3033A, Uscope 9.2F PU3022A (Zhuhai Pusen Medical Technology Co. Ltd. Guangdong, China), as well as the WiScope (OTU Medical Inc, CA, USA). To reflect real-world operating room situations, the single-use scopes (Pusen 7.5F, Pusen 9.2F and OTU WiScope) were brand new scopes from sealed packages. Reusable ureteroscopes (Storz and Olympus scopes) had all been rinsed and decontaminated after use in the operating theater, with no record of the number of previous interventions.

For the Storz Flex-X2s, the Power LED 175 light source (unit used < 100 h) was used with a corresponding 230 cm/3.5 mm fiber-optic cable, together with the IMAGE1 S HX-P HDTV 1-Chip pendular camera (Karl Storz SE & Co. KG, Tuttlingen, Germany). For the Olympus URF-P7 and URF-V3, the VISERA elite CLV-S190 light source (Xenon short-arc lamp used < 100 h) was used with a WA03310A 300 cm/4.3 mm fiber-optic light cable, as well as the CH S190 08 LB camera head (Olympus, Center Valley, PA, USA). Fiber-optic cables were entirely new.

A color spectrometer housing the Vishay VEML 6040 color sensor (RGBW200, ELV Elektronik AG, Leer, Germany) was used for lux (lx) measurements in saline, as previously described [13].

**Fig. 1:**
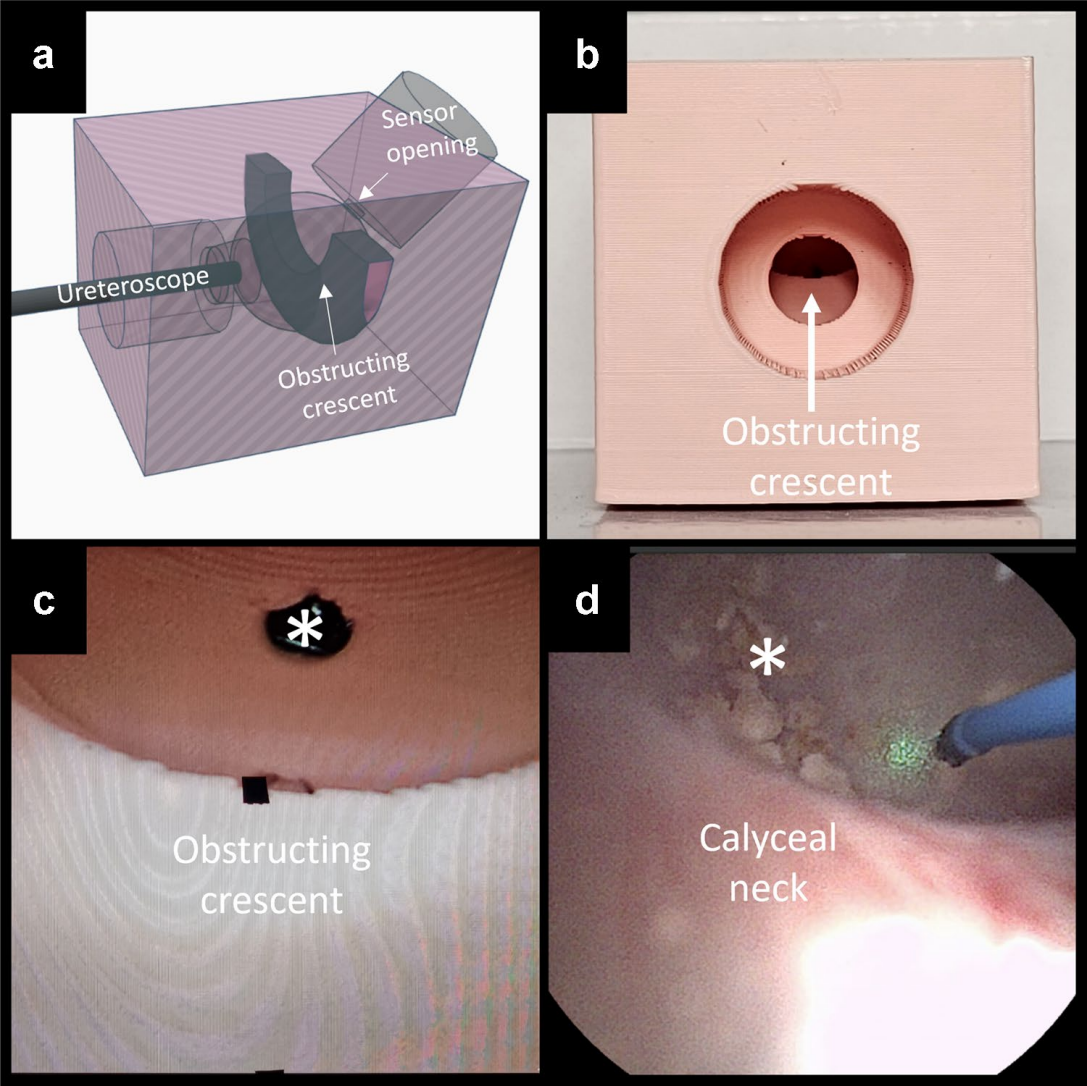
Experimental setup: obstructive kidney calyx model. **a** 3D visualization of the obstructive kidney calyx model from a side view. **b** Front view of the 3D-printed pink model. The large opening would fit a black rubber cylinder to hold the ureteroscope during experiments. To display the obstructive crescent within, the black rubber cylinder was removed in this photograph for the sake of better visualization. **c** Still image of the endoscopic view with the obstructive crescent situation at 6 o’clock. The spectrometer measurement opening is labeled with an asterisk (*). **d** Still image of an in vivo endoscopic view illustrating a typical clinical scenario where the light source of the endoscope was partially obstructed by the calyceal neck when trying to reach the target stone fragments (*) with the laser fiber (blue). The model of the current study was inspired from this clinical scenario

A 3D-printed pink obstructive kidney calyx model was used to hold the ureteroscopes at a fixed distance of 20 mm from the center of the opposite concave surface of the kidney calyx model in a dark room (Fig. 1). The model consisted of a closed spherical cavity replicating a human kidney calyx and included an obstructive crescent and a background illuminance measurement opening located at a 45°angle relatively to the axis of the scope. (Fig. 1a–c). The position was chosen to simulate a common occurrence in ureteroscopy with the target pathology being beyond the partially obstructing obstacle in the background (Fig. 1d). Pink was chosen for the kidney calyx model to replicate human urothelial mucosa. The ureteroscope was maintained in a straight position, with the center of the scope view aligned to the center of the opposite concave pink surface. The size of the target field and distance from the light sensor were chosen with reference to dimensions of models constructed on data from endocasts [15] used in studies testing scopes in the setting of laser lithotripsy [16, 17], to reflect in vivo settings. The obstructive crescent was shaped to mimic the calyceal neck when entering a kidney calyx.

All measurements were performed in saline to replicate the usual conditions found in clinical routine during ureteroscopy, in view of a previous study showing fundamental differences of ureteroscope illumination between air and saline [13].

For each obstructive situation, the sensor of the color spectrometer was placed at the 45° opening and measurements were repeated 5 times. The model was then turned in the axis of the scope to obtain measurements in four situations representing endoscopic calyx views with obstruction at 12, 3, 6, and 9 o’clock. For each repeated measurement, the ureteroscope was withdrawn and reinserted in the kidney calyx model.

Whenever the ureteroscope setup allowed for light brightness adjustment, measurements were repeated at light brightness settings of 50% and 100%. For the Storz Flex-Xc, this was found available to be adjusted only via the menu buttons on the scope handle. For the Storz Flex-X2s, in addition to brightness settings adjustable on the light stack, buttons on the camera head also allowed for separate brightness adjustment. Based on our findings with the Storz Flex-Xc, brightness setting for the Storz Flex-X2s in the camera head was kept at 50% and brightness settings only adjusted on the light source unit. For all other scopes, the brightness setting was adjusted on the light source unit only. Since light brightness can be set either to automatic or manual mode on the Olympus light source as well as with the Storz camera head, all measurements were performed using the manual mode to ensure consistent measurements throughout all experiments.

### Statistical analysis

Overall background illuminance was defined as mean from all 4 obstructive situations for a given scope. Background illuminance for a given obstructive situation was expressed as mean value of 5 repeated measurements. An analysis was performed for all scopes comparing inter-scope overall background illuminance, and inter-scope background illuminance for respective obstructive clock situations using one-way ANOVA with Tukey post hoc comparisons.

To quantify background illuminance variability for each scope, the obstructive clock situations for each scope were considered and reported as percentage relative to the highest in each scope (3 percentages for each scope). The highest relative percentage for each scope was defined as the intra-scope background illuminance variability, which reflects the range of background illuminance variance. Analysis with Student’s *T* test was carried out for all background illuminance variability values comparing scopes based on their tip design characteristics, as reported in a previous study [13]: 1 vs 2 light sources, position of working channel, and transparent vs non-transparent tips.

Intra-scope analysis for background illuminance comparing each obstructive situation was performed with one-way ANOVA.

For all tests, a two-sided *p* value < 0.05 was considered statistically significant. All statistical tests were performed with GraphPad Prism 10.0.2 (GraphPad Software, La Jolla CA, USA).

## Results

### Overall background illuminance

Overall background illuminance significantly differed between ureteroscopes (ANOVA *p* < 0.001 at both 50% and 100% brightness settings). These differences were as high as 40 times between scopes with the highest and lowest overall background illuminance, respectively (5897 lx vs. 146 lx, respectively) (Table 1). At 50% brightness setting, the highest overall background illuminance was found for the Flex-Xc (5897 lx), followed by the Flex-X2s (1977 lx), Pusen 9.2Fr (1037 lx), P7 (573 lx), V3 (411 lx), WiScope (259 lx) and finally lowest background illuminance with the Pusen 7.5F (146 lx). The order changed at 100% brightness setting with the V3 climbing two ranks: Flex-Xc (6059 lx), Flex-X2s (2727 lx), V3 (1799 lx), Pusen 9.2Fr (1684 lx), P7 (1429 lx), WiScope (505 lx), and finally Pusen 7.5F (266 lx).

**Table 1:** Comparison of mean background illuminance of flexible ureteroscopes in an obstructive kidney calyx model

Scope		Mean background illuminance at 50% brightness (lux) (95% CI)							Mean background illuminance at 100% brightness (lux) (95% CI)							Mean overall 50% vs 100% *p* value***
		Mean overall	Obstructive crescent situation relative to a clock face						Mean overall	Obstructive crescent situation relative to a clock face						
			12 o’clock obstruction	3 o’clock bstruction	6 o’clock obstruction	9 o’clock obstruction	*p* value*	Max. illuminance		12 o’clock obstruction	3 o’clock obstruction	6 o’clock obstruction	9 o’clock obstruction	p value*	Max. illuminance	
Reusable	Storz Flex-Xc	5897 (4708–7086)	3653 (3506–3800)	3208 (3052–3364)	8448 (8330–8566)	8279 (8011–8547)	*p* < 0.001	6 o’clock obstruction	6059 (4828–7289)	3636 (3285–3987)	3409 (3095–3723)	8452 (8176–8728)	8738 (7951–9524)	*p* < 0.001	9 o’clock obstruction	*p* = 0.84
	Storz Flex-X2s	1977 (1547–2407)	1371 (1336–1406)	1269 (1258–1281)	3488 (3322–3654)	1780 (1694–1866)	*p* < 0.001	6 o’clock obstruction	2727 (2156–3297)	1812 (1742–1882)	1835 (1815–1855)	4710 (4465–4955)	2550 (2373–2726)	*p* < 0.001	6 o’clock obstruction	*p* = 0.03
	Olympus V3	411 (307–514)	276 (247–306)	313 (305–320)	755 (536–974)	298 (251–345)	*p* < 0.001	6 o’clock obstruction	1799 (1386–2212)	1073 (1045–1101)	1276 (1215–1336)	3233 (2921–3545)	1616 (1448–1784)	*p* < 0.001	6 o’clock obstruction	*p* < 0.001
	Olympus P7	573 (535–610)	619 (599–639)	487 (466–507)	674 (656–693)	511 (502–519)	*p* < 0.001	6 o’clock obstruction	1429 (1281–1578)	1944 (1906–1981)	1212 (1208–1217)	1378 (1261–1495)	1184 (1135–1232)	*p* < 0.001	12 o’clock obstruction	*p* < 0.001
Single-use	Pusen 7.5F	146 (116–176)	91 (90–92)	94 (94–96)	244 (227–261)	154 (129–180)	*p* < 0.001	6 o’clock obstruction	266 (206–325)	148 (147–148)	154 (153–156)	427 (364–491)	333 (271–395)	*p* < 0.001	6 o’clock obstruction	*p* < 0.001
	Pusen 9.2F	1037 (870–1205)	764 (711–817)	627 (600–653)	1436 (1402–1471)	1322 (1270–1375)	*p* < 0.001	6 o’clock obstruction	1684(1401–1967)	1076 (1014–1139)	1120 (1095–1145)	2318 (2246–2390)	2223 (2140–2305)	*p* < 0.001	6 o’clock obstruction	*p* < 0.001
	OTU WiScope	259 (240–278)	242 (230–254)	225 (220–230)	246 (238–254)	323 (307–340)	*p* < 0.001	9 o’clock obstruction	505 (471–539)	456 (446–466)	435 (433–438)	510 (506–515)	617 (609–626)	*p* < 0.001	9 o’clock obstruction	*p* < 0.001
*p* value**		*p* < 0.001	*p* < 0.001	*p* < 0.001	*p* < 0.001	*p* < 0.001	–	–	*p* < 0.001	*p* < 0.001	*p* < 0.001	*p* < 0.001	*p* < 0.001	–	–	–

*Max*. maximum

*ANOVA comparing different positions at each 50% and 100% brightness settings

**ANOVA comparing across scopes for overall illuminance

*** Student’s *t* test comparing intra-scope overall illuminance 50% and 100% brightness settings

### Inter-scope comparisons

At each separate obstructive situation, background illuminance significantly differed between scopes (all ANOVA *p* < 0.001) (Table 1). Background illuminance variability across 50% and 100% brightness settings was the lowest with the WiScope (31%), followed by the P7 (39%), Pusen 9.2F (56%), Flex-Xc (62%), Flex-X2s (64%), Pusen 7.5F (65%) and finally highest variability with the V3 (67%) (Supplementary Table 1). For the V3, this translates to a background illuminance up to threefold higher when comparing the intra-scope obstructive situation with highest vs. lowest illuminance.

Scopes with transparent tips (P7 and WiScope) had significantly lower intra-scope background illuminance variability compared to scopes with non-transparent tips at 50% brightness setting (*p* = 0.01), but not at 100% brightness setting (*p* = 0.05). When grouped according to the number of light sources (2 vs. 1) and position of working channel (3 vs 9 o’clock), there were no significant differences in intra-scope background illuminance variability (all *p* > 0.3).

### Intra-scope comparisons

Intra-scope comparisons of overall background illuminance between 50 and 100% brightness showed significant differences for all scopes, except for the Flex-Xc (Table 1).

**Fig. 2:**
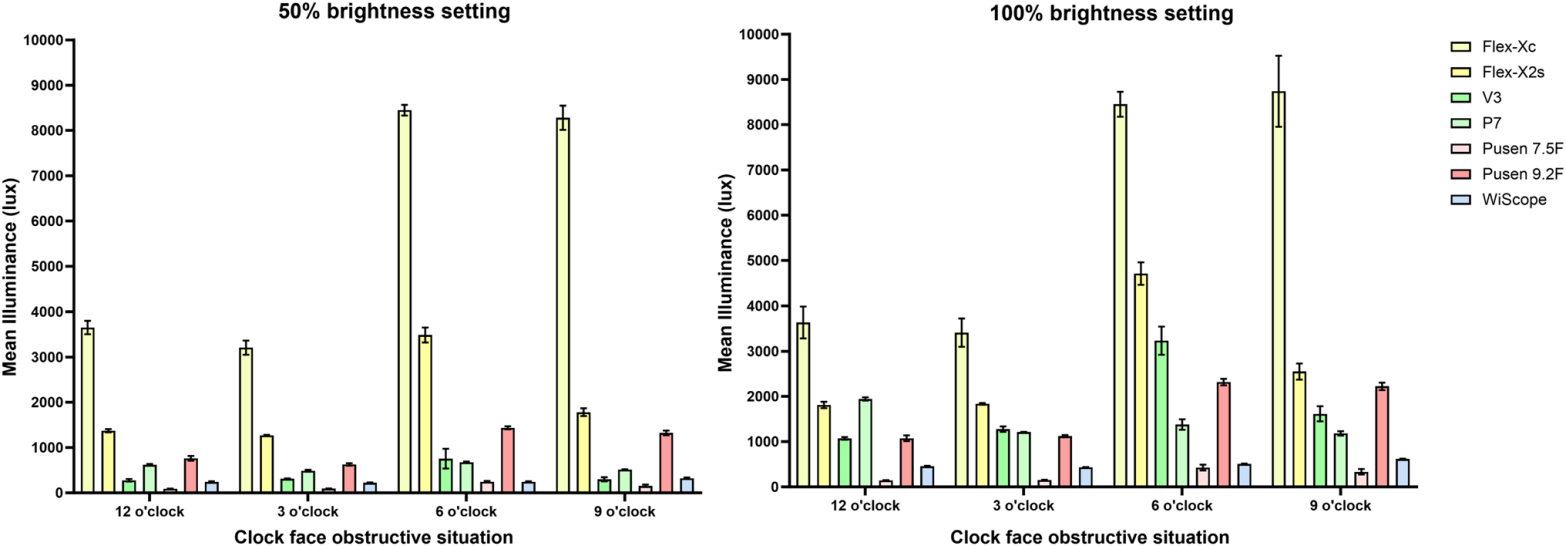
Background illuminance of ureteroscopes in different obstructive situations within a kidney calyx model. Mean background illuminance of ureteroscopes with different clock face obstructive situations in the in vitro model with whiskers indicating 95% CI

**Fig. 3:**
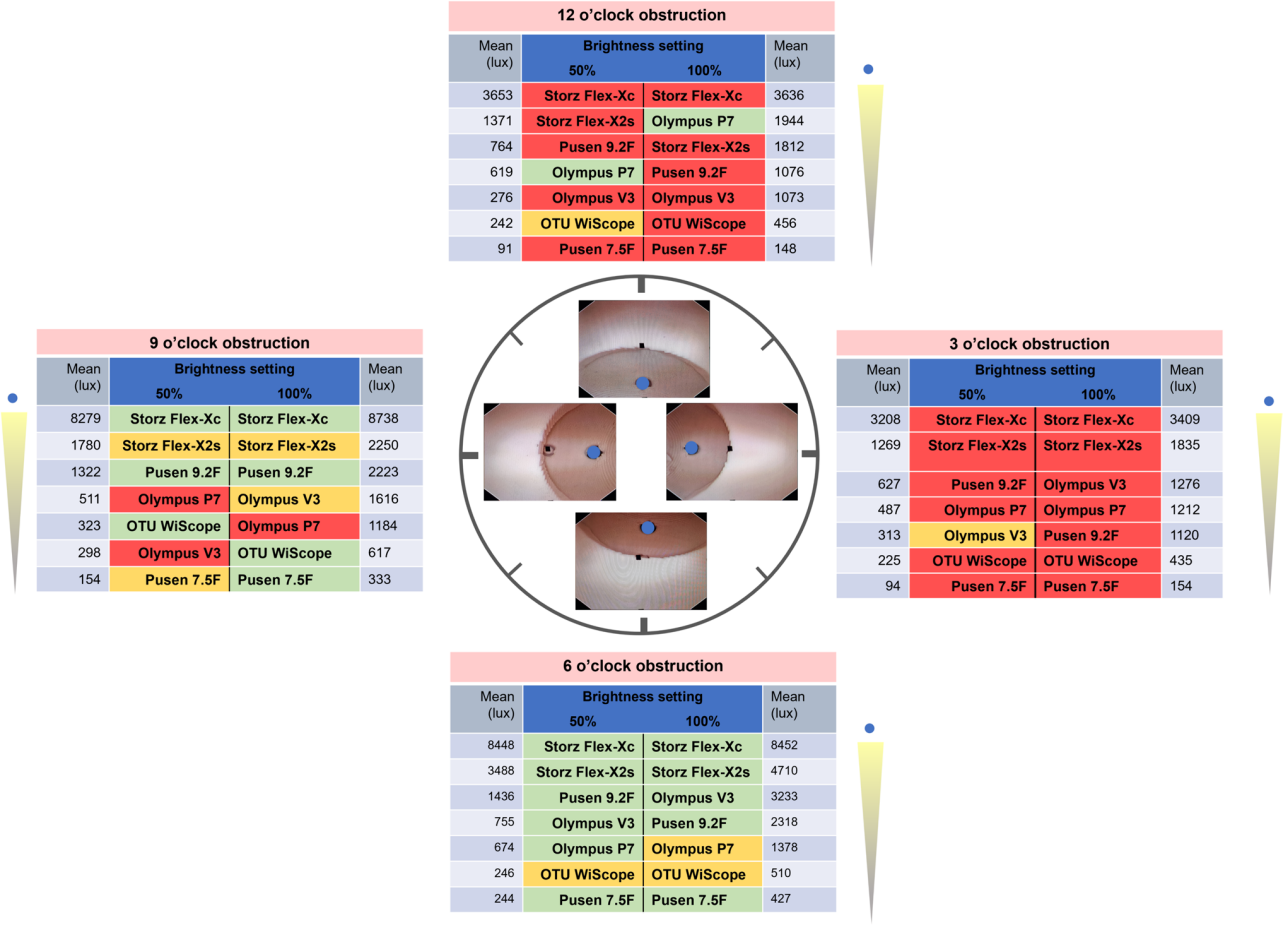
Background illuminance of ureteroscopes in different obstructive situations relative to a clock face (50% and 100% brightness settings). Mean background illuminance (lux) with each obstructive situation relative to a clock face. Ureteroscopes are ordered from highest to lowest illuminance for each situation. Images are still images from the experimental setup using a WiScope, and represent obstructive situations from 12, 3, 6, and 9 o’clock directions. Blue dots indicate the measurement opening position where the spectrometer sensor is placed. To highlight the intra-scope performance for each obstructive situation, color coding of the obstructive situations was done for mean background illuminance with each obstructive situation referenced to the 95% CI limits of the overall mean background illuminance for each given scope (green = higher than upper limit, orange = between upper and lower limit, and red = lower than lower limit). These limits are found in Table 1, first results column. Green coding represents best performance of a given scope, while red represents its worst performance

All scopes had the highest background illuminance with the 6 o’clock obstructive situation for the 50% brightness setting, except for the WiScope (9 o’clock obstructive situation) (Table 1, Fig. 2). This was the same for the 100% brightness setting except for the Flex-Xc (9 o’clock), WiScope (9 o’clock), and the P7 (12 o’clock). For the lowest background illuminance, at 50% brightness setting, this was found with the 3 o’clock obstructive situation for all scopes except for the V3 and Pusen 7.5F (12 o’clock). At the 100% brightness setting, this was with 12 o’clock for most scopes, except for the P7 (9 o’clock) and Flex-Xc/WiScope (3 o’clock). The obstructing situations that each scope performs better or worse in are shaded green and red, respectively, in Fig. 3.

## Discussion

Obstructive situations present a challenge to illuminance of ureteroscopes. This study confirmed that a selection of evaluated flexible ureteroscopes had different performance in the evaluated obstructive situations. The range of background illuminance variability differed largely between scopes, with the 3 o’clock obstructive situation (target at 9 o’clock) found with the greatest loss of background illuminance for most scopes.

A prior analysis of the same set of flexible ureteroscopes analyzed in a black open model [13] showed somewhat different results to the present study. Particularly, the directions of maximum illuminance in the black model did not correlate with the areas of higher background illuminance in this present study. Despite further accounting for the effects of direct and indirect light with a previous evaluation of illuminance skew within an enclosed kidney model [14], the obstructive situations in this present study also did not correlate entirely with the illuminance skew of the scopes from that study. Perhaps, this may be due to additional contributions from the center (non-peripheral) illuminance and indirect reflected light off the obstructing crescent in obstructive situations.

Our study found that the Flex-Xc had no significant difference of illuminance between 50 and 100% brightness, consistent with previous evaluations where adjustment of brightness settings on the Flex-Xc scope handle resulted in no change of actual illumination of the light source, but rather only increasing the exposure of the projected screen image [13, 14].

More interestingly, ureteroscopes with a transparent tip design were found to have significantly lower intra-scope illuminance variability when compared to their non-transparent tip counterparts. This suggests that ureteroscopes with a transparent tip are more versatile in obstructive situations, possibly due to allowance of direct and indirect light to pass through the transparent tip. The exact mechanisms and impact of transparent tip designs on ureteroscopy should be further evaluated.

**Fig. 4:**
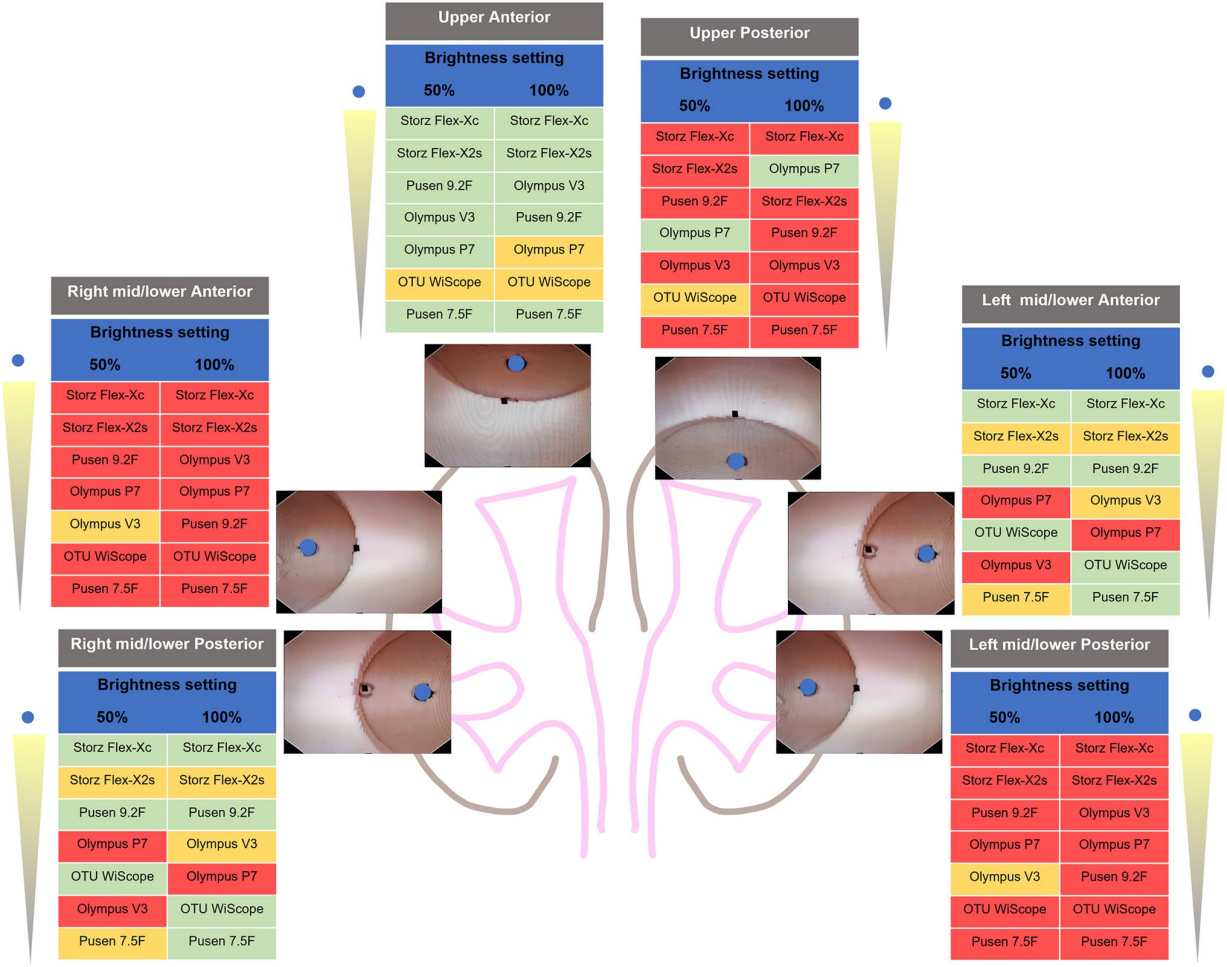
Background illuminance of ureteroscopes in different obstructive situations relative to typical calyceal locations. Mean background illuminance with each obstructive situation correlated to typical locations in a kidney. Ureteroscopes are ordered from highest to lowest illuminance for each situation. Images are still images from the experimental setup using a WiScope representing the various obstructive situations. Blue dots indicate the measurement opening position where the spectrometer sensor is placed. To highlight the intra-scope performance for each obstructive situation, color coding of the obstructive situations was done for mean background illuminance with each obstructive situation referenced to the 95% CI limits of the overall mean background illuminance for each given scope (green = higher than upper limit, orange = between upper and lower limit, and red = lower than lower limit). These limits are found in Table 1, first results column. Green coding represents the best performance of a given scope, while red represents its worst performance

To apply our findings clinically, the authors suggest that obstructive situations located at (a) 12 o’clock, (b) 3 o’clock, (c) 6 o’clock, and (d) 9 o’clock could typically occur in vivo when facing an (a) Upper posterior calyx, (b) Right mid/lower anterior and Left mid/lower posterior calyces, (c) Upper anterior calyx, and (d) Right mid/ lower posterior and Left mid/lower anterior calyces, respectively (Fig. 4). Our results show that most tested ureteroscopes have their best background illuminance in upper anterior, right mid/ lower posterior, and left mid/lower anterior calyces. On the other hand, most tested ureteroscopes had the worst background illuminance with the upper posterior, right middle/lower anterior, and left mid/lower posterior calyces.

Knowledge of the ureteroscope light distribution in obstructive situations can allow the surgeon to choose available scopes depending on laterality and pathology location (stones or tumors). This is akin to working channel location for the laser fiber when considering the left or right kidney [4]. Arguably, this can be overcome by turning the ureteroscope upside down and to use the flipped positions of the light distribution from the ureteroscope to the operator’s advantage, akin to turning the scope upside down to change the working channel direction during laser lithotripsy [18]. The interaction between area of maximum background illuminance and laser fiber position will additionally need to be further evaluated in future studies.

There are limitations that need to be acknowledged. First, the present study is an in vitro setting to assess ureteroscope properties that may impact in vivo surgery. The results of this study should be taken with prudence, since environmental factors may possibly affect clinical translation. Besides light obstruction, blood [19] and urine may additionally cause changes in illuminance characteristics, and this needs to be further explored in obstructive situations. While the obstructive situations were simulated to replicate real clinical conditions, there may possibly be other variations encountered in clinical routine. However, the obstructive situations in this present study can apply generally to most upper pole and mid/lower pole obstructive situations. Second, the reusable scopes were tested after usage in previous operations with possible deterioration of the optical systems (illumination and imaging [20]) with prior usage. This, in fact, contributes to the translation of our results to real-world conditions in endourology units using reusable scopes. Third, all measurements were done with the ureteroscope held straight, but there is arguably little change in illumination properties in the deflected state within the range specifications of each ureteroscope.

## Conclusions

Illuminance performance of ureteroscopes within an obstructed calyx model differed significantly for various obstructive situations. Across all obstructive situations, the Flex-Xc had the best background illuminance and the Pusen 7.5F had the worst. Urologists should be aware of the illuminance performance of scopes in different obstructive clinical situations to help guide their choice of ureteroscope.

## Supplementary Information

Below is the link to the electronic supplementary material.

Supplementary file1 (DOCX 16 KB)

## Data Availability

On request to corresponding author for raw data on the experimental setup.
