# Supplementary material for: Illumination matters Part III: Impact of light obstruction on illuminance from flexible ureteroscopes — a comparative PEARLS analysis
Source: World J Urol. 2024 Mar 23;42(1):188. doi: 10.1007/s00345-024-04910-9 (PMC10960769; doi:10.1007/s00345-024-04910-9)
Supplement: Supplementary file 1 — Supplementary file1 (DOCX 16 KB) [file 345_2024_4910_MOESM1_ESM.docx]

| **Supplementary table 1. Intra-scope background illuminance variability of flexible ureteroscopes in an obstructive kidney calyx model** | | | | | | | | | | | | | |
| --- | --- | --- | --- | --- | --- | --- | --- | --- | --- | --- | --- | --- | --- |
| Scope | | 50% brightness (lux) (95% CI) | | | | | | 100% brightness (lux) (95% CI) | | | | | |
|  |  | Max. illuminance obstructive situation | Obstructive situation vs max illuminance  (percentage change) | | | | Intra-scope variability | Max. illuminance obstructive situation | Obstructive situation vs max illuminance  (percentage change) | | | | Intra-scope variability |
|  |  |  | 12 o’clock  obstruction | 3 o’clock  obstruction | 6 o’clock  obstruction | 9 o’clock  obstruction |  |  | 12 o’clock  obstruction | 3 o’clock  obstruction | 6 o’clock  obstruction | 9 o’clock  obstruction |  |
| Reusable | Storz Flex-Xc | 6 o’clock | -57% | -62% | - | -2% | 62% | 9 o’clock | -58% | -61% | -3% | - | 61% |
|  | Storz Flex-X2s | 6 o’clock | -61% | -64% | - | -49% | 64% | 6 o’clock | -62% | -61% | - | -46% | 62% |
|  | Olympus V3 | 6 o’clock | -63% | -59% | - | -61% | 63% | 6 o’clock | -67% | -61% | - | -50% | 67% |
|  | Olympus P7 | 6 o’clock | -8% | -28% | - | -24% | 38% | 12 o’clock | - | -38% | -29% | -39% | 39% |
| Single-use | Pusen 7.5F | 6 o’clock | -63% | -61% | - | -37% | 63% | 6 o’clock | -65% | -64% | - | -22% | 65% |
|  | Pusen 9.2F | 6 o’clock | -47% | -56% | - | -8% | 56% | 6 o’clock | -54% | -52% | - | -4% | 54% |
|  | OTU WiScope | 9 o’clock | -25% | -31% | -24% | - | 31% | 9 o’clock | -26% | -29% | -17% | - | 29% |
| Max. = Maximum | | | | | | | | | | | | | |
